# Digitalisation anxiety: development and validation of a new scale

**DOI:** 10.1007/s44192-021-00003-w

**Published:** 2021-11-29

**Authors:** Katharina F. Pfaffinger, Julia A. M. Reif, Andreas K. Huber, Vera M. Eger, Melina K. Dengler, Jan Philipp Czakert, Erika Spieß, Rita Berger

**Affiliations:** 1 Ludwig-Maximilians-Universität München, Munich, Germany; 2 University of Barcelona, Barcelona, Spain

**Keywords:** Digitalisation anxiety, Scale development, ICT demands

## Abstract

The increasing spread of digital technologies and respective consequences for the way we live, work, and communicate can evoke feelings of tension and discomfort. This so-called digitalisation anxiety is related to existing and future technologies, includes the process of digitalisation in everyday life, and refers to multiple levels (the individual, organisations, and society). Existing scales measuring technology-related fears due not adequately reflect these features. Therefore, we developed the German version of the Digitalisation Anxiety Scale (DAS). Having generated items based on a qualitative interview study (Study 1, n = 26), we demonstrated the DAS’s factor structure, internal consistency and construct validity in Study 2a (n = 109) and test-retest reliability in Study 2b (n = 30). In Study 3 (n = 223), the scale’s structure was confirmed and correlates of digitalisation anxiety were examined. The final version of the DAS consists of 35 items with a four-factor structure (societal triggers for digitalisation anxiety, triggers related to interaction and leadership, triggers within oneself and triggers resulting from the digitalisation implementation process). Digitalisation Anxiety had negative relationships with well-being and performance. The scale allows practitioners and researchers to measure and benchmark individuals’ levels of digitalisation anxiety, and to track changes over time. The scale can inform interventions aiming at reducing digitalisation anxiety and stress resulting from digitalisation.

## Introduction

Alongside positive opportunities and chances, digitalisation goes along with new kinds of demands, which can evoke digitalisation anxiety. Digitalisation anxiety is defined as “feelings of tension and discomfort with respect to the emergence of new technologies and the integration of those technologies in all aspects of daily life” [1]. It is conceptualized as a predisposition, stable across different situations and time points [1]. Digitalisation anxiety can be triggered on the individual (e.g., self-imposed pressure, perceived loss of personal control due to digitalisation), organisational (increasing expectations placed on employees, e.g., regarding temporal availability), and societal (e.g., unpredictable consequences of digitalisation for the way people live and work) level [1].

Research has already dedicated to the conceptualization of negative feelings regarding technologies. For example, the concepts technology anxiety [2], information technology anxiety [3], technophobia [4–6], technostress [7, 8], or, more specifically, computer anxiety [9–12], computerphobia [13, 14], computer aversion [15], computer resistance [16], and barriers to the adoption of augmented reality [17] have been suggested. These existing concepts all comprise negative feelings (e.g., stress, anxiety, fear) related to the (anticipated) use of technology in general or specific types of technologies.

However, these constructs differ from digitalisation anxiety because they refer to existing technologies. They do not include *future technologies* which do not yet exist in vivo but are anticipated in sensu. Technological developments in recent decades range from the introduction of the first personal computers and their proliferation to the widespread adoption of the internet and ubiquitous computing (which describes a hyper connected world in which computational technology is basically everywhere) [18]. Those developments call for a construct that is applicable to all new technologies, even those which might not even have been developed yet, because anxiety is not limited to what exists but can also be directed towards the unknown.

Moreover, existing constructs are related to specific items or objects and do not include the *process of digitalisation’s penetration* into everyday life. However, digitalisation is an ongoing process of technology integration into daily life, not just a single object or one-time event. Thus, it is crucial to take a process perspective when conceptualizing digitalisation anxiety and to focus on the process of digitalisation as a societal megatrend which is missing in existing scales on negative technology-related feelings [4–6].

Finally, existing concepts do not sufficiently consider the *multilevel structure* of anxiety triggers. Research has shown that negative feelings about digitalisation are related to stressors on different levels [7, 19, 20]. Although the techno-insecurity subdimension of technostress and technophobia address some triggers on a societal level, these triggers are limited to the fear of being replaced in one’s job and other implementation processes. These scales do not address long-term consequences that affect not only oneself but also other people and society in general. A new scale should therefore consider anxiety triggers on different levels.

For these reasons, following scale development standards [21], we developed and validated the Digitalisation Anxiety Scale (DAS) which is applicable to contemporary and future technologies, considers the process of digitalisation, and captures different levels of anxiety.

## Psychometric criteria and a partial nomological network

### Validity and reliability: Hypotheses 1–4

*Convergent validity* is a basic requirement for the psychometric quality of a psychological scale. Evidence of convergent validity is given if there are substantial correlations between different instruments measuring a similar construct [22]. To assess the DAS’s convergent validity [23], we thus examined its correlations with related scales.

First, the information technology anxiety scale (ITAS) [3] assesses anxiety related to information technology. The ITAS was developed to expand the previous focus on computers (and related computer anxiety) to include information technology in general [3]. With our DAS we have made a logically similar extension, that is, an extension of the previous focus on information technology to digitalisation in general. As information technology is one, but only one aspect of digitalisation and the DAS covers a wider range of digitalisation-related anxieties, there should be medium to high positive correlations [24] between the DAS and the ITAS:

#### Hypothesis 1a

The correlations between the DAS and the ITAS are *r* ≥ 0.30.

Second, the techno-insecurity subdimension of the technostress scale (TINS) [8] was used to test the DAS’s convergent validity. The TINS focuses not just on the use of information and communication technologies (ICTs), but also assesses negative feelings towards ICTs on a more general level, including fear of risks such as job loss. It also contains a felt threat by other people who are supposed to have a better understanding of ICTs. These organisational and social issues are also targeted by the contents of the DAS, but on a more general level. Therefore, there should be medium to high positive correlations [24] between the DAS and the TINS. Accordingly, we suggest:

#### Hypothesis 1b

The correlations between the DAS and the TINS are *r* ≥ 0.30.

To show that digitalisation anxiety is distinguishable from other constructs, we tested the scale’s *discriminant validity* and examined DAS’s relationship with symptoms of generalized anxiety as measured in the Penn State Worry Questionnaire (PSWQ) [25]. Generalized anxiety is characterized by playing through catastrophe scenarios, whereby these scenarios are unspecific, that is, they cannot be narrowed down to a certain topic. Moreover, the focus of the catastrophe scenarios’ content changes frequently [26]. By contrast, within our construct, anxiety has a specific focus on digitalisation and has no clinical component. Both instruments should therefore clearly differentiate between people who are excessively and clinically worried about their future (PSWQ) and people whose (non-clinical) worries focus specifically on digitalisation (DAS). The relationship between the DAS and the PSWQ should thus be weak [24] as the scales measure conceptually different constructs:

#### Hypothesis 2

The correlation between the DAS and the PSWQ is *r* < 0.30.

We assessed *criterion validity* by testing whether the DAS predicted aversion of digitalisation with avoidance of and disliking digitalisation as practically relevant behavioural indicators. Behavioural avoidance is a correlate of anxiety [27]. Adapted to the specific context of digitalisation, behavioural avoidance could be expressed in an aversion of digitalisation, that is, in avoiding and disliking digitalisation:

#### Hypothesis 3

The DAS positively correlates, at least to a medium extent, r > 0.30, with digitalisation avoidance and disliking digitalisation*.*

As we conceptualized digitalisation anxiety as a stable predisposition, we expected *test–retest-reliability*:

#### Hypothesis 4

When measuring digitalisation anxiety at two consecutive points in time, the correlation between the results should be high, that is r > 0.50.

### Correlates of digitalisation anxiety: Hypotheses 5–7

To embed digitalisation anxiety in a nomological network of relevant related variables, we examined its relationships with well-being, recovery, and performance.

To analyse the relationship between digitalisation anxiety and *well-being*, we focused on both positive (satisfaction, engagement) and negative (stress, strain) aspects of well-being. According to “affect as information theories” (e.g., [28]), “affective states are primary to cognitive evaluations” in that “more intense, negative affective states will be accompanied by less favourable cognitive evaluations” [29]. Applying this logic to the context of our research, we assumed that digitalisation anxiety represents a negative affective state which is accompanied by a less favourable evaluation of the working situation, that is, reduced work-related satisfaction. Moreover, according to the job demands-resources model [30], job-related anxiety is negatively related to engagement, which, transferred to our context, implies that digitalisation anxiety should be negatively related to work engagement.

Regarding negative well-being indicators and building on the job demands-resources model [30], we assumed that people experiencing digitalisation anxiety would experience higher levels of stress and strain. The job demands-resources model explains this relationship with anxious people’s increased self-undermining behaviours which lead to higher levels of demands and consequently higher levels of strain. In sum, we propose:

#### Hypothesis 5

Digitalisation anxiety is negatively related to well-being.

To analyse the relationship between digitalisation anxiety and *recovery*, we focused on sleep quality and quantity, as well as on detachment. We built our reasoning on the stressor-detachment model [31]: Negative affect such as anxiety triggered by job stressors leads to higher levels of negative activation, which is not only present immediately after the encounter with the stressor, but may also holds at the end of the workday. This high negative activation stimulates recall of further negative events and experiences [32] and makes it more difficult for people to detach from work which also impairs sleep [33, 34]. Taken together we assume:

#### Hypothesis 6

Digitalisation anxiety is negatively related to recovery.

In an organisational context, it is not only important to consider employee well-being and recovery, but also employee *performance*, which is why we also investigated the relationship between digitalisation anxiety and the subjective performance indicators productivity and innovation. Attentional control theory [35], and its precursor, processing efficiency theory [36] explain how anxiety impairs performance in that efficient functioning of the goal-directed attentional system is impaired and worries pre-empt cognitive processing and storage capacity. Anxiety causes working memory deficits, alters retrieval from long-term memory, and narrows attention [37]. This basic idea is also taken up in the job demands-resources model [30], which suggests that employees suffering from strain (including work-related anxiety) “do not have the energetic resources to reach their work goals”. We therefore suggest:

#### Hypothesis 7

Digitalisation anxiety is negatively related to subjective performance ratings.

**Fig. 1:**
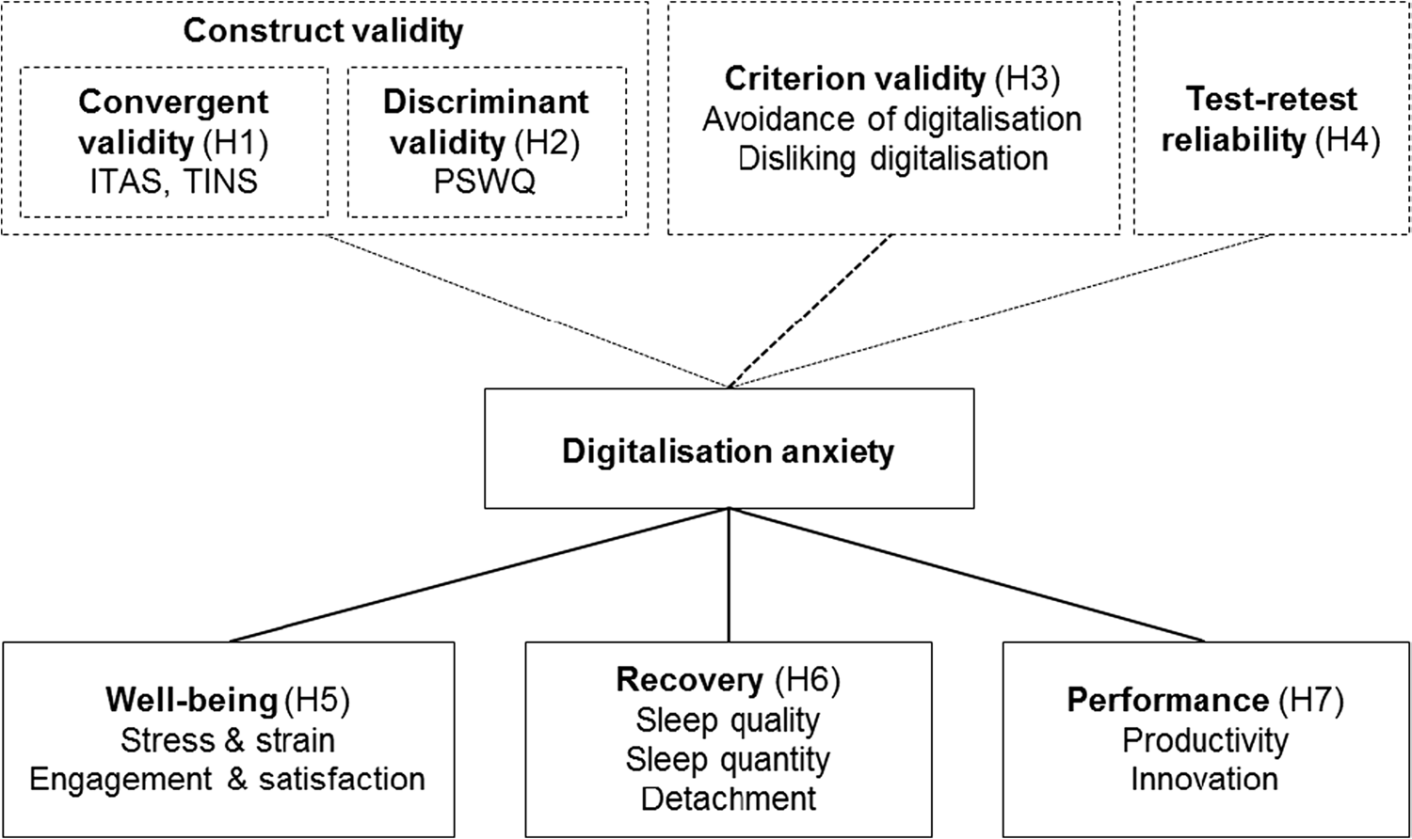
Overview of nomological network and hypotheses. *ITAS* information technology anxiety scale, *TINS* techno-insecurity subscale of technostress scale, *PSWQ* Penn state worry questionnaire, *H* hypothesis

Corresponding effects have been found for similar constructs describing negative feelings related to digitalisation or technology [2, 7, 10, 38]. For an overview of all hypotheses see Fig. 1.

## Scale development and validation

Table 1 gives an overview of studies in the scale development and validation process. All studies were carried out in accordance with the Ethical Principles of Psychologists and Code of Conduct by the American Psychological Association [39] and with the Declaration of Helsinki [40]. Participants provided informed consent.

**Table 1:** Overview of studies in the scale development and validation process

Study		Development and validation process
1	Item development	
	Qualitative interviews (n = 26) Expert ratings (n = 3) Cognitive interviews (n = 4)	Item generation Content adequacy assessment Questionnaire assessment
2a	Structure exploration, consistency and validity	
	Quantitative survey (n = 109)	Exploratory factor analysis Internal consistency analysis Construct validity
2b	Reliability over time	
	Quantitative survey (n = 30)	Test–retest reliability
3	Structure confirmation and correlations	
	Quantitative survey (n = 223)	Confirmatory factor analysis Correlates of digitalisation anxiety

### Study 1: item development

#### Method

In Study 1, as part of a larger research project (see funding information), 26 employees (male: n = 13, female: n = 11, no gender indicated: n = 2; age: *M* = 43.1 years, no age indicated: n = 2) were interviewed with a semi-structured interview guide on their hopes and fears associated with digitalisation to generate items. The transcribed interviews were used to formulate items on digitalisation anxiety in this Study. (The data were also used by [1] to identify triggers of digitalisation anxiety). In order to ensure content adequacy, ratings by three experts were used to confirm that the developed items covered all three levels of digitalisation anxiety triggers (societal, organisational, and individual) [1, 20]. To test the comprehensibility of the items, we conducted cognitive interviews with four additional persons, who had not been involved in the interviews or expert ratings (age: *M* = 35.50 years; gender: male: *n* = 2, female: *n* = 2) and asked them to think out loud while reading the items and answering them.

#### Results

Based on the interview data, we generated 73 items. According to the expert ratings, the items adequately covered the triggers of digitalisation anxiety [1, 20]. The results of the cognitive interviews showed that four items were not comprehensible, nine items had a similar content, and the syntax of two items was too complex. We adapted or excluded the respective items, resulting in 67 items that covered the suggested digitalisation anxiety trigger levels.

### Study 2a: structure exploration, consistency and validity

In Study 2a, we explored our scale’s factor structure and examined its internal consistency and construct validity.

#### Method

A total of 109 employees completed an online survey (male: n = 44, female: n = 65; age: *M* = 33.11 years, *Min* = 18, *Max* = 67). Employment was a prerequisite for participation. The mean working time was 27.74 h per week (*SD* = 13.77, *Min* = 4, *Max* = 50). Participants worked in different sectors (industry: n = 12, services: n = 37, public administration: n = 4, education: n = 22, health: n = 13, other: n = 20, no information: n = 1).

*Digitalisation Anxiety* was measured with the 67 preliminary items developed in Study 1. Within this set of 67 items, no reversed items were included to avoid an artificial factor structure [41]. DAS items were answered on a 6-point Likert scale (1 = *strongly disagree*, 6 = *strongly agree*).

Apart from these items developed to measure digitalisation anxiety, the following scales and items were included in the questionnaire: We used 16 items from the PSWQ [25] to measure g*eneralized anxiety* (e.g., “I am always worried about something”) and to test the scale’s discriminant validity. Items were answered on a 5-point Likert scale indicating how typical the items are for oneself (1 = *not at all typical for me*; 5 = *very typical for me*). Moreover, we used 12 items from the ITAS [3] to measure *information technology anxiety* (e.g., “Working with IT would make me very nervous”; 1 = *strong disagreement*; 7 = *strong agreement*) and 5 items from the TINS [8] to measure *techno-insecurity* (e.g., “In my current job I am continuously feeling threatened by new technologies”). TINS items were answered on a 5-point Likert scale (1 = *strong disagreement*; 5 = *strong agreement*), with a sixth option for participants without an opinion (6 = *no opinion*). With the ITAS and the TINS we wanted to test the scale’s convergent validity.

As a behavioural indicator to test criterion validity, we measured *aversion of digitalisation* with the items “I avoid digital technologies at work when possible” (avoidance) and “I do not like dealing with topics concerning digitalisation” (disliking). Avoidance and disliking were answered on a 6-point Likert scale (1 = *not at all*; 6 = *to a great degree*).

All items were presented in German language. English items were translated following established translation-back-translation procedures [42]. For Cronbach’s alphas see Table 4.

#### Results

Conducting exploratory factor analysis (EFA), and using parallel analysis [43] and the scree plot, we identified four factors of the DAS. To select items for the final scale, we descriptively evaluated the range of answers, mean scores, factor loadings (highest factor loading should be > 0.40 and > 2 × second highest factor loading of item, side loadings should be ≤ 0.30), communalities (should be ≥ 0.40) and distribution of answers (there should not be two modes) [44], which resulted in a final set of 35 items. Table 2 shows factors and their Cronbach’s alphas which were good to very good for all factors. Table 3 shows the factor loadings, mean values, standard deviations, and communalities after extraction of the final scale. Table 4 shows the correlations between the DAS and the other scales and indicators.

**Table 2:** Factor description and assignment to digitalisation anxiety trigger levels

Factor	Factor description	Assignment to anxiety trigger levels by [1]	Number of items	Cronbach’s alpha
1	General digitalisation anxiety	Societal level	15	0.94
2	Self-related digitalisation anxiety	Individual level	8	0.94
3	Interaction- and leadership-related digitalisation anxiety	Organisational level	7	0.88
4	Implementation-related digitalisation anxiety	Organisational level	5	0.83
Full scale			35	0.96

**Table 3:** Items, descriptives, factor loadings, and communalities resulting from the EFA

Item (English version)	Item (German version)	Factor loadings				*M*	*SD*	*C*
		1	2	3	4			
I am concerned about digital systems not being secure enough	Es bereitet mir Sorgen, dass digitale Systeme nicht sicher genug sind	**0.840**	− 0.270	− 0.079	0.054	4.21	1.42	0.435
I am afraid that humanity will become dependent on technology due to digitalisation	Es macht mir Angst, dass die Menschheit infolge der Digitalisierung von Technologie abhängig wird	**0.812**	− 0.041	0.059	− 0.092	4.00	1.64	0.598
As a result of digitalisation I am increasingly afraid of hacker attacks	Infolge der Digitalisierung habe ich zunehmend Angst vor Hacker-Angriffen	**0.801**	− 0.242	0.115	− 0.172	4.01	1.42	0.413
I am afraid that surveillance will increase due to digitalisation	Mir macht es Angst, dass die Überwachung durch die Digitalisierung zunimmt	**0.794**	0.069	− 0.279	− 0.021	4.18	1.58	0.467
I am afraid that in a digital world technology will be used against humans	Ich habe Angst, dass in einer digitalisierten Welt Technologie gegen den Menschen eingesetzt wird	**0.773**	− 0.027	0.100	− 0.050	3.61	1.61	0.632
I am afraid of a lack of control due to digitalisation	Ich habe Angst vor einem Kontrollverlust infolge der Digitalisierung	**0.721**	0.285	− 0.175	− 0.007	3.38	1.62	0.694
I am concerned about how the increasing amount of data due to digitalisation will be used	Mir bereitet es Sorgen, wie die durch Digitalisierung steigende Menge an Daten genutzt wird	**0.687**	0.135	− 0.130	− 0.089	4.08	1.61	0.441
I am afraid of a new extent of criminality which is made possible by the use of digital technology	Ich habe Angst vor einem neuen Ausmaß an Kriminalität, das durch den Einsatz digitaler Technologien ermöglicht wird	**0.676**	− 0.260	0.263	− 0.058	4.08	1.47	0.437
I am concerned that the human working force will be replaced due to digitalisation	Es macht mir Angst, dass die menschliche Arbeitskraft infolge der Digitalisierung ersetzt werden könnte	**0.628**	0.149	0.222	− 0.259	3.15	1.47	0.621
I am concerned about digitalisation as it entails consequences on many aspects of life	Mir bereitet die Digitalisierung Sorgen, weil sie Auswirkungen auf viele Bereiche des Lebens hat	**0.564**	0.265	0.113	− 0.044	3.26	1.62	0.698
I am concerned about the human needs not being taken into account sufficiently in the implementation of digitalisation	Ich mache mir Sorgen, dass die Bedürfnisse des Menschen bei der Umsetzung der Digitalisierung nicht ausreichend berücksichtigt werden	**0.548**	0.056	0.007	0.201	4.05	1.53	0.530
I am afraid that people will trust technology more than humans due to digitalisation	Es macht mir Angst, dass infolge der Digitalisierung der Technologie mehr vertraut wird als Menschen	**0.529**	− 0.058	0.281	0.026	3.40	1.53	0.509
I am afraid of a too strong trust in the proper functioning of technology in a digitalised world	Mir macht es Angst, dass in einer digitalisierten Welt zu sehr auf das Funktionieren der Technik vertraut wird	**0.498**	− 0.007	0.174	00.151	3.94	1.43	0.514
I am afraid of digitalisation as I see risks in the technological progress	Ich habe Angst vor der Digitalisierung, weil ich Risiken im technologischen Fortschritt sehe	**0.475**	0.246	0.074	0.126	3.06	1.51	0.652
I am afraid of the society being controlled by artificial intelligence due to digitalisation	Ich habe Angst, dass die Gesellschaft infolge der Digitalisierung von künstlicher Intelligenz gesteuert wird	**0.444**	0.110	0.181	0.036	2.72	1.52	0.471
I worry that I won’t be able to keep up due to digitalisation	Ich befürchte, dass ich selbst durch die Digitalisierung nicht mehr mithalten kann	− 0.194	**1.092**	0.005	− 0.172	2.33	1.34	0.811
I worry that I will be overwhelmed by the developments in the digitalised world	Ich befürchte, dass ich von den Entwicklungen in der digitalisierten Welt überfordert werde	− 0.100	**1.088**	− 0.002	− 0.153	2.51	1.43	0.910
I am afraid that I won’t be able to understand new processes in the digital world	Ich habe Angst, dass ich neue Prozesse in der digitalen Welt nicht verstehe	− 0.168	**1.039**	0.064	− 0.077	2.59	1.42	0.872
I am concerned that I am expected to quickly understand new processes in the digital world	Mir macht es Sorgen, dass von mir erwartet wird, neue Prozesse in der digitalen Welt schnell zu verstehen	− 0.031	**0.806**	0.196	− 0.088	2.57	1.39	0.782
I am skeptical about the use of digital technology at work	Ich stehe dem Einsatz neuer digitaler Technologien bei meiner Arbeit skeptisch gegenüber	0.016	**0.639**	0.131	− 0.005	2.47	1.46	0.552
I am afraid of digitalisation as I feel helplessly exposed to it	Ich habe vor der Digitalisierung Angst, weil ich mich dieser hilflos ausgesetzt fühle	0.107	**0.578**	0.228	− 0.022	2.28	1.40	0.677
I worry that digitalisation will not facilitate my work	Ich befürchte, dass die Digitalisierung meine Arbeit nicht erleichtert	0.050	**0.537**	− 0.005	0.152	2.94	1.53	0.437
I worry that digital technology is not user friendly	Ich befürchte, dass digitale Technologie nicht benutzerfreundlich ist	− 0.046	**0.503**	0.160	0.143	2.71	1.29	0.451
I am afraid that a robot could be my next coworker due to digitalisation	Es macht mir Angst, dass infolge der Digitalisierung ein Roboter mein nächster “Kollege” sein könnte	0.046	− 0.004	**0.739**	− 0.118	2.05	1.39	0.518
I am afraid of being personally blamed for technical problems	Ich habe Angst, für technische Probleme persönlich verantwortlich gemacht zu werden	− 0.081	0.029	**0.692**	0.118	2.45	1.46	0.518
I am feeling anxiety about the future due to digitalisation as I perceive a threat to my workplace due to it	Mir bereitet die Digitalisierung Zukunftsängste, weil ich meinen Arbeitsplatz dadurch bedroht sehe	− 0.263	0.269	**0.566**	0.064	1.99	1.21	0.408
I worry that I will face communication problems due to digital communication	Ich befürchte, dass ich durch die digitale Kommunikation Verständigungsprobleme haben werde	− 0.080	0.217	**0.550**	0.025	2.44	1.41	0.451
I am afraid that the work of humans will be less valued as a result of digitalisation	Ich habe Angst, dass die Arbeit von Menschen infolge der Digitalisierung weniger wertgeschätzt wird	0.259	− 0.097	**0.523**	0.106	3.03	1.44	0.502
I am afraid that I will be other-directed by technology due to digitalisation	Ich habe Angst, dass ich infolge der Digitalisierung von Technik fremdbestimmt werde	0.288	0.030	**0.488**	0.090	2.93	1.47	0.615
I am afraid of being replaced by younger and better educated employees due to digitalisation	Ich habe Angst, infolge der Digitalisierung von jüngeren, besser ausgebildeten Mitarbeitern ersetzt zu werden	0.077	0.244	**0.447**	− 0.051	2.17	1.34	0.448
I am afraid that there is no sound concept for the implementation of digitalisation	Mir macht es Angst, dass es kein gutes Konzept für die Umsetzung der Digitalisierung gibt	− 0.029	− 0.009	− 0.125	**0.933**	3.63	1.50	0.751
I am afraid that many questions related to digitalisation have not been clarified yet	Mir macht es Angst, dass viele Fragen der Digitalisierung noch nicht geklärt sind	0.231	0.002	− 0.055	**0.661**	3.54	1.56	0.617
I am concerned about the appropriate education of future generations in a digital world	Ich mache mir Sorgen um die passende Ausbildung zukünftiger Generationen in der digitalen Welt	0.014	0.012	0.111	**0.576**	3.36	1.54	0.422
I am concerned about digitalisation as employees are not incorporated in the changes	Die Digitalisierung bereitet mir Sorgen, weil Mitarbeiter in die Veränderung nicht miteinbezogen werden	0.077	− 0.034	0.140	**0.530**	3.48	1.35	0.405
I worry about the occurrence of chaos due to digitalisation	Ich befürchte, dass durch die Digitalisierung ein Chaos entsteht	0.135	0.185	0.070	**0.447**	3.00	1.45	0.500

Method of extraction: Maximum Likelihood; Method of rotation: Promax with Kaiser-normalization; Rotation is converged in 8 iterations; C = Communalities after extraction; Only items of the final scale are shown in the table; Descriptives, factor loadings, and communalities refer to the German version of the DAS

**Table 4:** Correlations between the DAS and other scales and indicators

	DAS	PSWQ	ITAS	TINS	Avoidance	Disliking
DAS	*0.963*					
PSWQ	0.255**	*0.761*				
ITAS	0.725**	0.328**	*0.834*			
TINS	0.329**	0.047	0.294**	*0.815*		
Avoidance	0.526**	0.216*	0.698**	0.309**		
Disliking	0.486**	0.252**	0.618**	0.153	0.643**	

Numbers in diagonal indicate Cronbach’s α of the scales (if more than 1 item)

The correlations between the DAS and the ITAS as well as the TINS were both higher than 0.30 which provides evidence for the convergent validity of the DAS and supports Hypothesis 1. The correlation between the DAS and the PSWQ was smaller than 0.30, which supports Hypothesis 2 and provides evidence for the discriminant validity of the DAS. Both behavioural indicators (avoidance, disliking) were significantly positively related to DAS, providing evidence for the scale’s criterion-oriented validity and supporting Hypothesis 3.

### Study 2b: reliability over time

In order to examine the reliability of the DAS over time, we calculated the test–retest correlation coefficient in Study 2b.

#### Method

Thirty participants (male: n = 6, female: n = 23, no gender indicated: n = 1, age: *M* = 31.87, *Min* = 19, *Max* = 59) answered the DAS in an online survey both at the beginning of the study and after 13 days. (Between the two measurement points, participants answered three items on stress, satisfaction, detachment, and work every 2 days in the scope of a student research project).

#### Results

The test–retest correlation for the DAS at time 1 and time 2 was *r* = 0.84, which indicates stability over time and is above Post’s [45] suggested cut-off for acceptable test–retest reliability. This result supports Hypothesis 4.

### Study 3: structure confirmation and correlations

In Study 3, we assessed the adequacy of the scale’s structure by conducting a confirmatory factor analysis (CFA). We also examined correlations of digitalisation anxiety with relevant constructs.

#### Method

A total of 223 employees (male: n = 92, female: n = 121, diverse: n = 2, no information: n = 8; age: *M* = 33.02 years, *Min* = 18 years, *Max* = 68 years, no information: n = 10) took part. Participants worked in different positions (employee: n = 160, self-employed: n = 8, part-time student worker: n = 27, intern: n = 4, university student assistant: n = 10, other: n = 6, no information: n = 8) and different sectors. Participants reported a mean regular working time of 31.57 h per week. They used ICTs at work for 20 h per week on average and for work-related purposes at home for 6.41 h per week on average. A total of 207 employees who reported a working time of at least 10 h per week were included in the calculation of digitalisation anxiety’s correlates as being employed was a prerequisite.

We measured *digitalisation anxiety* with the 35 items of the DAS (Cronbach’s α = 0.97, e.g., “I am concerned about digital systems not being secure enough”).

To capture *well-being*, we measured stress and strain with 10 items according to Haslam and Reicher [46] (Cronbach’s α = 0.87; e.g. “I feel exhausted”, 1 = *not at all*, 5 = *to a very great degree*) and engagement and satisfaction with 6 items according to [47] (Cronbach’s α = 0.84, e.g., “So far I have achieved all my goals at work”, 1 = *not at all*, 5 = *to a very great degree*).

To capture *recovery*, we measured sleep quality with 4 items (Cronbach’s α = 0.78; e.g., “How often in the past month did you have trouble falling asleep?” [48]), which were answered on a 6-point Likert scale indicating the frequency of sleep problems in one month, 1 = *22–31 days*, 2 = *15–21 days*, 3 = *8–14 days*, 4 = *4–7 days*, 5 = *1–3 days*, 6 = *never*), with higher values indicating a higher quality of sleep. Sleep quantity was measured with the item “How many hours of sleep did you get on average per night in the last week?” [49]. Detachment was measured with 4 items (Cronbach’s α = 0.89, e.g., “I forget about work” [50]) answered on a 5-point Likert scale (1 = *do not agree at all*, 5 = *fully agree*).

To capture *performance*, we measured self-rated (subjective) productivity related to ICTs with 4 items (Cronbach’s α = 0.92, e.g., “Information and communications technologies help to improve the quality of my work” [8]) and innovation with 4 items (Cronbach’s α = 0.85, e.g., “I’m coming up with new ideas at work” [51]). Both were answered on a 5-point Likert scale, 1 = *do not agree at all*, 5 = *fully agree*.

As ICT use, gender, and age have been found to affect well-being and sleep quality [52], we included them as control variables. Items were presented in German language.

#### Results

**Fig. 2:**
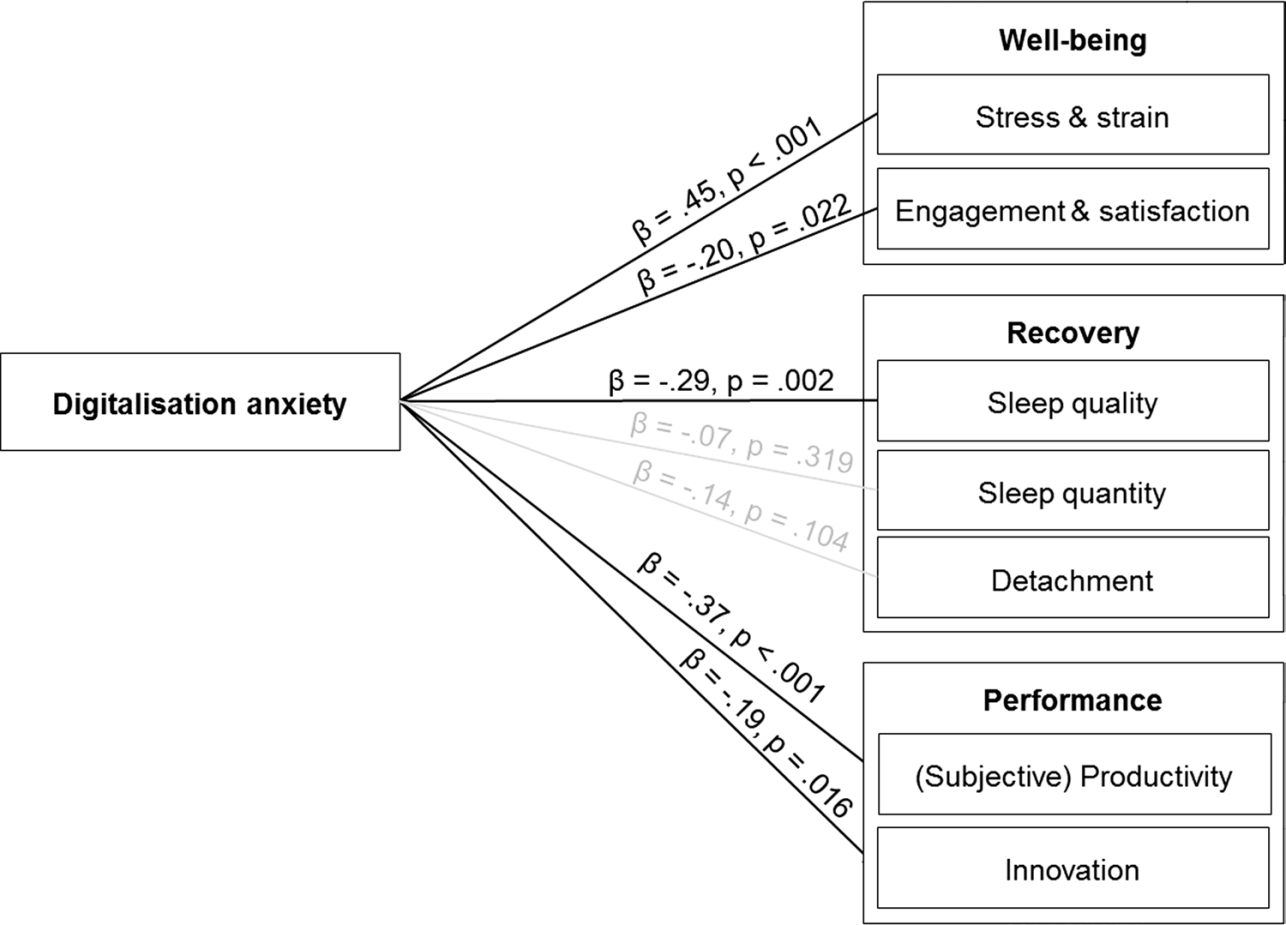
Structural equation model (SEM) with relationships between digitalisation anxiety and well-being, recovery and performance indicators*.* βs represent standardized coefficients. Control variables are not depicted for better clarity of the results. Age is negatively related to sleep quality, gender is positively related to sleep quantity (coding of gender variable: 0 = male, 1 = female), working hours are negatively related to productivity, overtime is positively related to stress & strain and negatively related to sleep quality, and ICT use at work and at home are positively related to productivity

We calculated confirmatory factor analysis (CFA) to test the factor structure of the DAS. Our model, χ^2^ (542) = 1015.92; p < 0.001; ratio χ^2^ to degrees of freedom: 1015.92/542 = 1.87; RMSEA = 0.064, CFI = 0.910, TLI = 0.902, SRMR = 0.060, exhibited an acceptable fit when allowing for correlated error terms (applying thresholds suggested by Fuglseth and Sørebø [53]). To examine the relationships between digitalisation anxiety and the well-being, recovery, and productivity indicators (Hypotheses 5, 6, and 7), we calculated a structural equation model (SEM) using the software RStudio (Version 1.1.453). We included age, gender, ICT use, technostress inhibitors, and language as control variables. Digitalisation anxiety was positively related to stress and negatively related to engagement and satisfaction (support for Hypothesis 5). Digitalisation anxiety was negatively related to sleep quality. The relationships with hours of sleep and detachment were not significant, providing only partial support for Hypothesis 6. Digitalisation anxiety was negatively related to subjective productivity and innovation (support for Hypothesis 7) (Fig. 2).

## General discussion

The digitalisation anxiety scale (DAS), developed and validated in the German language, consists of 35 items with a four-factor structure: a general factor with societal triggers for digitalisation anxiety (15 items), a factor for triggers related to interaction and leadership (7 items), one factor with triggers within oneself (8 items) and one factor for triggers resulting from the digitalisation implementation process (5 items). The scale exhibited a high internal consistency and ratings were stable over time. Digitalisation anxiety measured with the DAS was distinct from generalized anxiety (PSWQ) and negatively related to well-being, recovery (sleep quality), and self-rated performance.

### Theoretical implications

The DAS extends existing work on technology-related fears and stress in the following ways: First, the DAS is not related to specific technologies and therefore is also applicable to future, anticipated technologies. The items refer to digital technology, digital communication, digital systems or digitalisation in general, showing that technologies that people are not yet used to or do not yet exist might particularly cause anxiety. Second, the DAS addresses digitalisation as an ongoing process involving the integration of technology into all aspects of daily life. This process perspective is reflected in two ways: (a) The DAS includes a unique subscale describing anxiety triggers related to the implementation of technologies and digitalisation. (b) Items are formulated in a way that incorporates a process perspective, mostly by using verbs such as “become” or “increase”, which describe processes or developments. Third, the three-factor structure by Pfaffinger et al. [1] was quantitatively replicated and integrated in the DAS. In addition, we further differentiated the structure by splitting the organisational factor into an implementation factor and an interaction and leadership factor, which describe two distinct organisational aspects. Taken together, we did not only take an existing scale on technology related fears and change the focus of its existing items on a more general phenomena, as for example reported by [2] and [3]. Instead, we explored the phenomenon of digitalisation anxiety in a substantive way and conceptualized it based on qualitative and quantitative data.

### Practical implications

As digital technologies are increasingly penetrating our daily lives, attitudes and fears towards these technologies should be continuously monitored with effective measures. The DAS scale can be used as such a measure by managers or supervisors to identify the “top triggers” of digitalisation anxiety within an organisation or by individuals to detect their own top triggers. Completing the DAS can help organisations and individuals develop measures to counteract the identified worries. Our studies revealed that digitalisation anxiety is related to behavioural indicators, potentially providing evidence of a vicious cycle: Digitalisation anxiety is related to avoidance behaviour, which makes it hard to have positive experiences related to digitalisation that might decrease one’s perceived level of digitalisation anxiety. The differentiation into digitalisation anxiety levels might point to possible ways to stop this vicious cycle by specifically intervening either on levels with less digitalisation anxiety or purposefully targeting levels with high digitalisation anxiety in order to achieve the greatest possible impact and help employees cope with their greatest fears and worries.

### Future research

In the interviews, participants also mentioned positive aspects and expectations regarding digitalisation, and those statements could be a starting point for conceptualizing a positive counterpart to digitalisation anxiety, such as technology readiness [54]. Other scholars also described possible fears related to the absence of digitalisation or technical devices such as “nomophobia” as fear of being without a mobile device [4, 55]. Future research could investigate individual and situational characteristics that affect whether digitalisation or the absence of it evokes negative feelings. As we did not examine third variables as moderating or mediating mechanisms in our study, we cannot make any statements about digitalisation anxiety’s mechanisms of effect. Future research could investigate technostress inhibitors [7] as moderating variable. Antecedents of digitalisation anxiety could also be tested in future studies. Wang et al. [56] found that the extent of power centralization in an organisation is positively related to the level of employee technostress, which might also hold true for digitalisation anxiety.

### Limitations

Although each individual study’s sample was small, we found empirical support for the DAS’s validity and reliability in three distinct, diverse samples in terms of occupation, age, and gender. Nevertheless, further confirmatory validation replicating the findings with larger samples would be desirable. Although the scale was originally developed and validated in German with a German sample, an English version of the scale has also been provided, which can be used to conduct a validation study with an English-speaking sample. Study 3 examining external validity was a cross-sectional study. Consequently, we were not able to make statements about the causal effects of digitalisation anxiety. Longitudinal designs should be applied to provide insights into the causal relationships between digitalisation anxiety and its correlates.

## Conclusion

Many concepts and scales for technology-related fears and stress exist. However, the DAS, based on qualitative interviews, covers current concerns independently of previously existing scales. The DAS takes a process perspective on digitalisation, measures anxiety triggers on different levels, and incorporates the possibility that one might fear things that are still unknown.

## Data Availability

The datasets generated during and/or analysed during the current study are available from the corresponding author on reasonable request.

## Code availability

Codes are available from the corresponding author on reasonable request.
